# Porcine Reproductive and Respiratory Syndrome Surveillance in breeding Herds and Nurseries Using Tongue Tips from Dead Animals

**DOI:** 10.3390/vetsci8110259

**Published:** 2021-11-02

**Authors:** Jordi Baliellas, Elena Novell, Vicens Enric-Tarancón, Carles Vilalta, Lorenzo Fraile

**Affiliations:** 1 Grup de Sanejament Porcí, 25192 Lleida, Spain; jordi@gsplleida.net (J.B.); elena@gsplleida.net (E.N.); vicens@gsplleida.net (V.E.-T.); 2 Upnorth Analytics, Arbeca, 25140 Lleida, Spain; cvilalta@upnorth.es; 3 Agrotecnio Center, 25198 Lleida, Spain; 4 Departament de Ciència Animal, ETSEA, University de Lleida, 25198 Lleida, Spain

**Keywords:** PRRSV, monitoring, tongues exudate, swine

## Abstract

The detection capacity of Porcine Reproductive and Respiratory Syndrome virus (PRRSV) in tongues from dead animals in breeding herds (stillborns and piglets dying during the lactating period) and nursery farms (naturally dead animals) for PRRSV surveillance was evaluated. The samples were selected if pairs of serum and tongues were available from 2018 to 2020. Serum (pools of five) and exudate from tongues (one bag) were analyzed by PRRSV RT-PCR. The agreement between the serum sample procedure versus tongues exudate was assessed using a concordance test (Kappa statistic) at batch level. A total of 32 submissions, corresponding to 14 farms, had PRRSV diagnostic information for serum and tongues exudate. The overall agreement of batch classification as positive or negative, based on RT-PCR PRRSV results, between serum and tongue exudate of the 32 pairs was 76.9%. Cohen’s Kappa was 0.55. The main discrepancy came from the presence of positive samples in tongues exudate and not in serum, suggesting that tongue exudate to monitor PRRSV seems to be more sensitive than serum. These results suggest that this sample procedure could be also used for PRRSV surveillance and monitoring.

## 1. Introduction

Porcine reproductive and respiratory syndrome (PRRS) is the most prominent endemic pig viral disease that continues to generate huge economic losses to producers [1]. PRRS virus (PRRSV) diagnosis is commonly based on clinical suspicion followed by laboratory confirmation with diagnostic procedures such as RT-PCR and antibody detection with an enzyme-linked immunosorbent assay (ELISA) technique. Clinical suspicion becomes evident if a sudden increase in the abortion rate and/or a relevant decrease in the number of born alive piglets and/or an increase of lost piglets (stillborn and mummified) is observed in sow farms [2]. These clinical symptoms are commonly observed after the beginning of the PRRSV outbreak in breeding herds, but their severity usually decreases as the prevalence of infectious breeding sows declines over time and these animals clear the infection [3,4]. Nevertheless, the losses associated with this disease could still be relevant for producers due to continuous virus recirculation during the nursery and fattening period after controlling the disease in the sow herd [5].

Several management strategies to control PRRSV are based on implementing a gilt acclimation protocol, a PRRSV vaccination program for gilts and sows, and a specific program to control the spread of pathogens in suckling pigs [6] including the possibility of vaccinating these animals [7] and the implementation of restrictive biosecurity measures in the farrowing room. On the other hand, eradication methods are mainly based on test and removal, herd depopulation and repopulation, herd closure and rollover at the farm level, and PRRSV elimination at a regional level [8]. In any case, knowledge of the epidemiological status from the sows to the fattening pigs is critical to adopt the most suitable measures adapted to each production system [4].

A lot has been learnt about the epidemiology of PRRS due to the continuous monitoring of breeding herds [9,10]. Until recently, a breeding herd was classified as “positive stable” after four consecutive negative tests on due-to-wean piglet sera collected every 30 days (or more frequently) over a 90-day period, testing in pools of five [11]. A minimum of 30 serum samples was required at each sampling in order to provide 95% confidence to detect a prevalence ≥10% [11]. A shortcoming of this approach is that PRRSV can persist in breeding herds at a prevalence of <10% as the disease evolves in the sow population [12,13,14]. Thus, PRRSV can sustain low-prevalence infection in breeding herds in subclinically infected herds and illustrate the need for improved surveillance methods and monitoring protocols. For instance, the latest American Association of Swine Veterinarians PRRS classification guidelines, published in September 2021, addressed this issue by increasing the number of due-to-wean piglets to be sampled [15]. Moreover, there are still unanswered questions regarding the role of nurseries and finishers in PRRSV epidemiology, which comprise most of the swine population (~90%).

One of the main issues regarding the surveillance and monitoring of large populations of animals is how to sample them in a cost-effective way. Oral fluid (OF) testing has recently emerged as a cost-effective alternative for monitoring several pig diseases. Thus, OF can be routinely collected in a ‘welfare-friendly’ way by any individual with a high probability of detecting PRRSV in infected herds [16,17]. Recently, the use of processing fluids recovered at the time of castration and tail docking in US breeding herds has also been proven to be a sensitive tool that allows veterinarians and producers to sample large numbers of animals at processing in a cost-effective way [18]. For instance, collecting tails and testicles in a Ziploc bag and testing the resultant fluids yielded a sensitivity value over 80% at the litter level [19]. Castration is no longer allowed in Europe for animal welfare reasons. On the other hand, preliminary results in breeding herds suggest that the use of removed tails could be a sensitive approach to define PRRSV status at the litter and herd level [20]. However, tail docking is also a controversial topic in the European Union and should be avoided unless tail biting problems appear at a farm level. An alternative to sampling live animals would be monitoring dead animals. The presence of dead animals in pig production is a common event across the production system; sampling carcasses does not require special skills, and it can be carried out by any person available on the farm without compromising animal welfare standards. Therefore, the main objective of this pilot study was to evaluate the detection capacity of the aggregation of removed parts from dead animals (tongues) as an indicator of PRRSV in swine farms, particularly in breeding herds and nursery farms, to provide additional surveillance options for producers.

## 2. Materials and Methods

### 2.1. Samples

The samples were selected from the ones submitted to the Grup of Sanejament Porci (GSP) for PRRSV diagnosis between July 2018 and December 2020. Briefly, the GSP is a non-governmental organization integrated with independent swine producers, cooperatives, and pig integration companies (http://www.gsplleida.net/es (accessed on 11 October 2021). The GSP runs a Veterinary Diagnostic Laboratory focused on swine diseases and offers coverage to a representative part of the Spanish pig population. The criteria for sample selection were: (1) Several positive PRRSV RT-PCR results of serum and tongues exudate from the same farm, (2) pairs of samples (serum vs. tongues) must have been collected from the same batch, and (3) the number of serum samples collected was at least 30 in order to detect a 10% of prevalence with a 95% of confidence level [11]. Moreover, two PRRSV-naïve farms were also included to have negative samples either in serum or tongues exudate. Finally, the time between sampling and diagnosis of PRRSV outbreak for each farm was recorded. Moreover, modified live-vaccine PRRSV was applied neither in piglets nor in sows during the last third of gestation in the included farms.

### 2.2. Sample Collection Protocol

**Figure 1:**
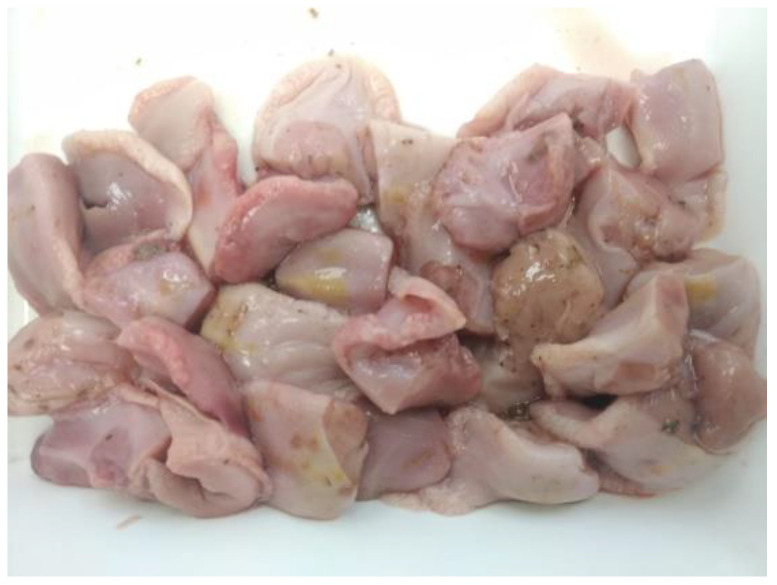
A representative sample of thawed tongue tips following the collection procedure.

A protocol for tongue collection was sent to all the producers to proceed in the same way in all the cases. Samples were removed from stillborns and piglets dying during the lactation period in breeding herds or from naturally dead piglets in nurseries. Briefly, two centimeters of the tip from the tongues (Figure 1) were removed and placed into a clean and new re-closable bag. To facilitate the collection, it was recommended that tongue tips were collected before disposing the daily dead animals. Tongues from stillborn and piglets dying during the lactation period, coming from the same farrowing batch, were aggregated in different bags in breeding herds. The number of tongue tips per bag submitted was between 30 and 100 piglets, depending on the farm size and mortality. In nurseries, it was recommended to aggregate the tongues of dead animals for one week. Bags were allocated in a freezer at −20 °C until submission to the laboratory. Cleaning and disinfection of the cutting instruments were recommended before and after the daily tissue collection to avoid any contamination between samplings. Tissue bags were sent to the laboratory in isolated containers either together with serum samples or alone.

Finally, thirty serum samples were collected from poor-health piglets during the same weeks that tongues from naturally dead piglets of the same farrowing batch in breeding herds. Similarly, thirty poor-health piglets amongst the nursery population were sampled the same week as tongue collection from naturally dead animals. Serum was collected using a single-use sterile needle and collection tube for each animal. Serum samples were submitted in the following 24 h after collection and sent to the laboratory under refrigeration.

### 2.3. Diagnostic Testing

**Figure 2:**
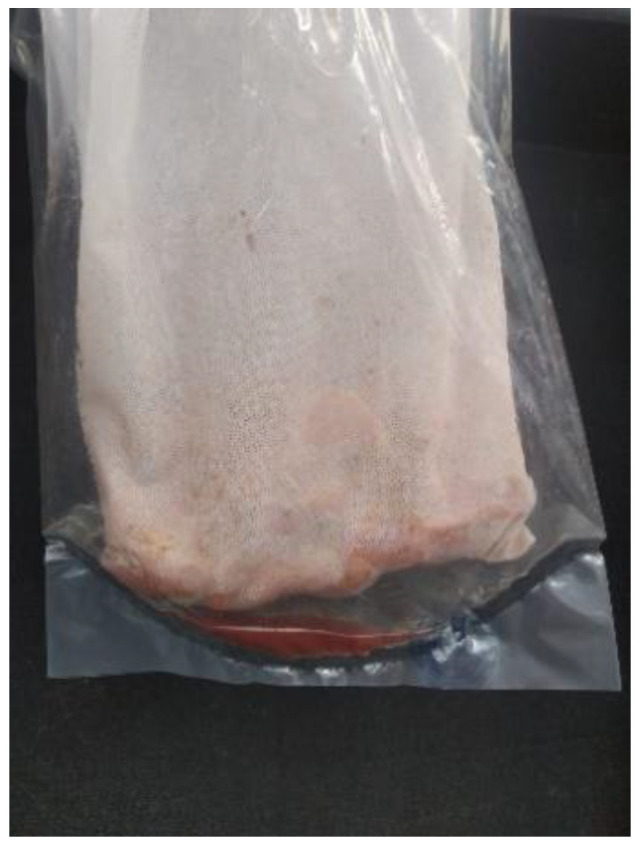
Resultant exudate at the bottom of the bag after homogenizing the sample with the paddle blender. Exudate was collected with a sterile syringe and placed in a sterile container for testing.

Serum samples were tested in pools of five as previously recommended by Holtkamp et al. 2011 [11]. Each bag of tissue was thawed, placed in a laboratory paddle blender (Stomacher^®,^ Seward Ltd., Worthing, West Sussex, UK), and homogenized for 60 s at medium speed. The exudate at the bottom of the bag was collected using a sterile syringe and placed in a sterile container for testing (Figure 2). Serum and exudate were analyzed by PRRSV RT-PCR according to the manufacturer’s recommendations. Briefly, total RNA was isolated from serum or exudate using the LSI™ MagVet™ Universal Isolation Kit (Thermo Fisher Scientific Inc., Waltham, MA, USA) in accordance with the manufacturer’s instructions. An internal positive control “IPC PRRS” was included within each sample and extracted according to manufacturing instruction to validate RNA extraction step. Samples were analyzed with LSI™ VetMAX™ PRRSV European (EU) and North American (NA) strains (EU/NA) Kit (Life Technologies, Thermo Fisher Scientific Inc., Waltham, MA, USA). Viral RNA was amplified as a one-step reverse transcriptase (RT)-PCR reaction, according to the kit instructions. Each 25 µL-reaction contained 7 µL of RNA and 18 µL of Mix PRRS European (EU) and North American (NA) strains (EU/NA) from the kit. The RT-PCR reactions were carried out on a 7500 Fast Real-Time PCR System, laptop, QST (Life Technologies, Thermo Fisher Scientific Inc., Waltham, MA, USA) in a 96-well format according to the manufacturer’s recommendations (10 min at 45 °C, 10 min at 95 °C followed by 40 cycles of 15 sec denaturation at 95 °C and 70 sec annealing at 60 °C). A sample was considered positive if the RT-PCR PRRSV Ct value was equal to or below 35. Moreover, the open reading frame 5 region (ORF5) of all positive serum samples were sequenced using Sanger technology as previously described [21] and some sequences coming from tongue exudate (from farms 4, 50, 36, 6, 9, and 52) were also sequenced for this study.

### 2.4. Data Analysis

The agreement between the serum sample procedure versus tongue exudate was assessed using a concordance test (Kappa statistic) at batch level and the total agreement determination [22] using publicly available software http://www.winepi.net/uk/index.htm (accessed on 1 October 2021).

All sequences were aligned using the Multiple Sequence Comparison by Log-Expectation (MUSCLE) algorithm in Geneious^®^ 10.0.7 (Biomatters, Ltd., Auckland, New Zealand) with default settings to compare and highlight variations across the sequences. A similarity matrix with all the sequences collected was built and compared with the database of sequences of the laboratory. Finally, maximum likelihood (ML) phylogenetic trees were constructed using RAxML software (https://directory.fsf.org/wiki/RAxML, accessed on 1 October 2021). One reference strain available in GenBank https://www.ncbi.nlm.nih.gov/genbank/ (accessed on 1 October 2021) (Lelystad PRRSV strain) was also included in both trees and similarity matrixes.

## 3. Results

A total of 32 submissions, corresponding to 14 farms, had PRRSV diagnostic information for serum and tongue exudate that fit the selection criteria. A total of 25 out of 32 submissions of matching specimens corresponded to dead piglets sampled in the farrowing room (stillborns and piglets dying during the lactation period) and seven were from dead piglets sampled in the nursery. A detailed description of the farm and samples characteristics can be found in Table 1 and Table 2.

The timing from the PRRSV outbreak diagnosis at a sow farm to sampling was from 15 to 464 days in the breeding herds and from 64 to 464 in nursery farms. The overall agreement of batch classification as positive or negative, based on RT-PCR PRRSV results, between serum and tongues exudate of the 32 pairs was 76.92% (95% confidence interval (CI): 60.73–93.12%) and the Cohen’s Kappa was 0.55 (95% CI: 0.23–0.87).

**Figure 3:**
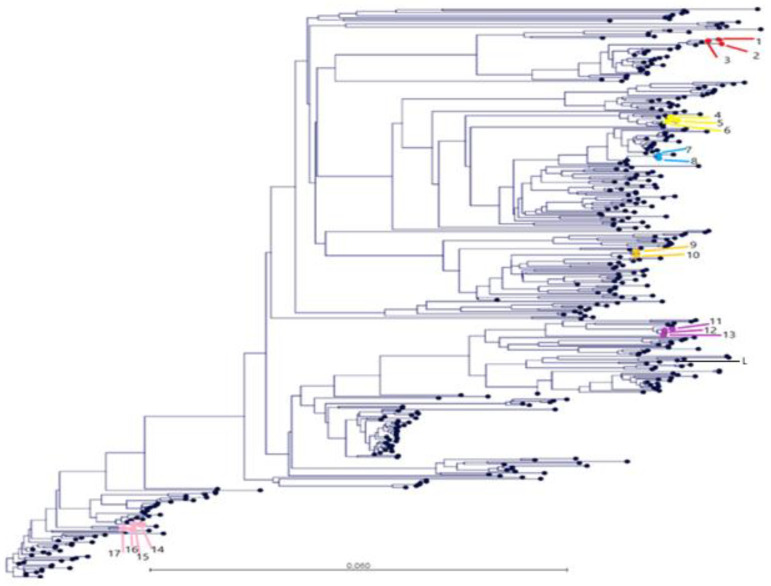
Phylogenetic tree of the Porcine Reproductive and Respiratory Syndrome virus (PRRSV) open reading frame 5 region (ORF5) of the different strains obtained from the Grup of Sanejament Porci (GSP) from serum samples between 2018 and 2020 and one reference strain (Lelystad PRRSV strain) available in GenBank (L in the figure). Some sequences coming from tongue exudate were also sequenced for farms 4, 50, 36, 6, 9, and 52 in this study. Samples coming from farms 4 (14,15,**16**,17), 50 (1,**2**,3), 36 (4,5,**6**), 6 (7,**8**), 9 (9,**10**) and 52 (**11**,12,13) are represented in pink, red, light yellow, blue, dark yellow, and violet, respectively. The identification number of the sequences are detailed in brackets by farm and highlighted in bold if the origin is tongue exudate.

Phylogenetic trees were constructed with PRRSV ORF5 sequences obtained from the GSP database during the period 2018–2020, which included the sequences from this study and one reference strain available in GenBank (Lelystad PRRSV strain). As detailed in Figure 3, the sequences obtained from farms 4, 36, 6, 9, 52, and 50 in the farrowing room clustered together independently if they were obtained from serum or tongue exudate. Thus, the nucleotide similarity was between 98.8 and 99.8% for each included farm across the study period. It deserves to be highlighted that sequences from farm 4 clustered together independent of the timing of sampling, type of sample (tongue exudate or serum), and the site of virus detection (nursery and farrowing farm). The nucleotide similarity for farm 4 samples ranged between 99.3 and 99.8% across the study period.

## 4. Discussion

This study described the process of collection of aggregated tissues (tongues) coming from dead animals as an alternative method for monitoring PRRSV presence in pig production systems. Serum has been widely accepted as reference sample to monitor the presence of PRRSV across a pig production system. Thus, the protocol based on sampling 30 piglets at weaning provided 95% confidence of finding ≥1 positive animal(s) in a large (>1000 pigs) population with a prevalence of ≥10% in sow farms using serum [11]. In our case, total agreement of tongues exudate with serum was 76.9% and the concordance (kappa value) between both samples was moderate (0.55). Thus, differences exist between the results obtained from serum versus tongue exudate. Nevertheless, the main discrepancy came from the presence of positive samples in tongue exudate and not in serum (Table 1 and Table 2) because only one case was positive in serum and negative in tongues exudate. Thus, tongue exudate to monitor PRRSV seems to be more sensitive than serum to detect PRRSV at the farm level. One of the reasons for this different detection by sampling carcasses could be based on targeting a subpopulation of animals (stillborn or dead piglets) that are more likely to harbor PRRSV. This reason would be especially important in low-prevalence scenarios wherein the sample size to detect low prevalence (<10%) could be unaffordable from an economical and practical point of view [23]. The farm PRRSV prevalence could not be determined because the main goal was to detect the presence of the virus in the herd. However, it is probable that this prevalence could be below 10% in many included farms due to the long period of time between PRRSV diagnosis and sampling (>240 days in many cases). Thus, the use of carcass parts offers a more cost-effective alternative to serum for monitoring PRRSV in large populations of pigs, either in the farrowing room or in the rearing phase. Additionally, sampling animals with a higher likelihood of harboring the virus could help to determine the true status of virus circulation in the herd.

We have been able to detect PRRSV after a long period of time since PRRSV diagnosis in sow and nursery farms using tongues of dead animals. Furthermore, similar ORF5 sequences matching the previously diagnosed were found. Our results agree with other published studies. Thus, 3 out of 825 sows were PRRSV PCR positive on serum 15 months after the initiation of a PRRSV test-and-removal elimination project in one farm [13]. In another farm, PRRSV was detected by virus isolation in 1 out of 60 sows (1.7%) two years after a PRRSV outbreak [14]. The introduction of PRRSV-naïve animals provides the opportunity for PRRSV to re-establish itself in the herd when the prevalence is non-zero; however, it was not possible to detect it with the standard diagnostic approach. Thus, PRRSV can be sustained at low prevalence in breeding herds over time, suggesting that the currently accepted protocols may not be adequate for detecting the virus under low-prevalence scenarios [12,13,14]. Increasing the sample size to detect low PRRSV prevalence would be one way to deal with the uncertainty of PRRSV detection, as suggested per the latest American Association of swine veterinarians (AASV) PRRSV classification guidelines [15]. Moreover, the clustering (non-random distribution) of positive litters within farrowing rooms would also require a large sample size to achieve detection [24]. In this sense, sampling tissues of dead animals is a way to focus on sampling high-risk animals and, therefore, increase the probability of PRRSV detection.

The selection of tongues as target tissue in carcasses was initially based on the amount of exudate that could be extracted after thawing and paddle blender processing. Thus, 10 tongues could provide enough exudate for analysis (laboratory internal data). Another option would have been to use tails, but the amount of exudate obtained was extremely low without adding PBS (laboratory internal data). This issue was described previously in different studies comparing the sensitivity of litter-aggregated castration tissues (tails and testicles) to correctly classify a litter as PRRSV positive by RT-PCR compared to serum [19,20]. Thus, Vilalta et al. (2018) [19] reported that no noticeable fluid was coming out of the tails. In a second study, 10 mL of PBS were added to the bag containing all the tails from the litter and compared with the serum results. In that study, it was suggested that adding less PBS could improve the sensitivity of the specimen. There are more reasons to select tongue rather than tail exudate. Thus, the tongue exudate was cleaner than tail exudate because tails coming from carcasses were frequently contaminated with feces, especially when diarrhea occurred in the farm of origin. Finally, the diagnostic laboratory preferred the specimen containing tongues because saliva and amniotic liquid traces was part of the sample coming from stillborn piglets. Thus, the resultant exudate after the freeze–thaw cycle and paddling process was a mixture of blood, saliva, and amniotic fluid in stillborn piglets. There is an extensive body of literature reporting the PRRSV shedding pattern through saliva and the use of oral fluids (OF) to monitor and detect PRRSV [25]. Therefore, it seems reasonable to assume that the presence of saliva and/or amniotic liquid may increase the likelihood of PRRSV detection.

One key question that may arise is the potential advantages of using aggregated samples from dead animals versus OF. We have not carried out a specific study to compare in parallel OF versus carcass samples. However, OF samples should be refrigerated or frozen in a short period of time; otherwise, the RNA may lose some integrity [25]. Another potential problem of OF is the presence of polymerase chain reaction (PCR) inhibitors that may interfere with the test and yield either false-positive or false-negative results [26]. Finally, incorrect sample handling and the presence of inhibitors would have an impact on the quality of the ORF5 sequence, either giving an incomplete sequence or no sequence at all. We have not observed any of the former limitations using tongue exudates. In particular, the identification of PRRSV strains in tongue exudates allow us to obtain high quality sequences to follow up epidemiological studies.

Clinical field samples were obtained from farms, which include breeders and post-weaning farms. The ORF5 sequences for farm 4 were poorly divergent and clustered together in the tree, supporting that the movements of carrier animals from sow herds to nursery and from nursery to finishing appeared to be the main route of infection in an integrated product system, as recently published [27]. Similar results were obtained by Linhares et al., 2014 [14] in a longitudinal study of 56 herds that detected a virus that matched the open reading frame (ORF)-5 sequence of the “original” herd virus in five herds (8.9%) after failing to detect PRRSV in serum samples from pre-weaning piglets over 90 days. Thus, the proposed surveillance method, based on tongue exudates, could be used to carry out epidemiological studies for PRRSV at a farm, company, and regional level.

## 5. Conclusions

The use of carcass parts to monitor the presence of porcine reproductive and respiratory syndrome virus is a suitable method, even in low-prevalence scenarios for breeding herds and nurseries. Moreover, the sequences obtained from these samples allow to carry out sequencing for molecular epidemiological studies.

## Figures and Tables

**Table 1:** Sow farm and sample characteristics to study the agreement between paired serum and tongue samples (between brackets) for Porcine Reproductive and Respiratory Syndrome virus (PRRSV) diagnosis in a production batch.

Farm (Pair)	Farm Size ^1^	Type of Farm ^2^	PRRSV History ^3^	Age ^4^	Sample Date	Timing from Diagnosis to Sampling	PRRSV Batch Results Serum/Tongues	Agreement (Y/N) ^5^
4 (1)	2500	FTF	Positive	3 weeks	5 February 2019	371	−/+	N
4 (2)	2500	FTF	Positive	1 day	5 February 2019	371	−/+	N
4 (3)	2500	FTF	Positive	1 day	12 March 2019	406	+/+	Y
4 (4)	2500	FTF	Positive	3 weeks	12 March 2019	406	−/+	N
4 (5)	2500	FTF	Positive	1 day	9 May 2019	464	−/+	N
4 (6)	2500	FTF	Positive	1 day	28 July 2020	910	+/+	Y
5 (1)	2300	FTF	Positive	1 day	12 March 2019	464	+/+	Y
5 (2)	2300	FTF	Positive	1 day	28 July 2020	206	+/+	Y
6 (1)	1700	FTF	Positive	1 day	6 March 2019	15	+/+	Y
6 (2)	1700	FTF	Positive	3 weeks	6 March 2019	15	+/+	Y
8 (1)	2400	FTF	Positive	3 weeks	19 February 2019	120	−/+	N
8 (2)	2400	FTF	Positive	1 day	19 February 2019	120	+/+	Y
9 (1)	2350	FTF	Positive	1 day	11 March 2020	109	+/+	Y
9 (2)	2350	FTF	Positive	3 weeks	11 March 2020	109	+/+	Y
24 (1)	3000	FTF	Positive	1 day	16 July 2020	87	−/+	N
24 (2)	3000	FTF	Positive	1 day	18 June 2020	59	+/+	Y
36 (1)	2200	FTW	Positive	1 day	28 July 2019	203	+/+	Y
36 (2)	2200	FTW	Positive	1 day	18 September 2019	255	+/+	Y
47 (1)	750	FTW	Positive	1 day	10 September 2019	369	+/+	Y
50 (1)	2000	FTF	Positive	1 day	9 December 2020	330	+/+	Y
50 (2)	2000	FTF	Positive	1 day	3 November 2020	294	+/+	Y
50 (3)	2000	FTF	Positive	1 day	8 September 2020	238	+/+	Y
52 (1)	3080	FTF	Positive	1 day	18 August 2020	120	+/+	Y
54 (1)	650	FTF	Negative	1 day	25 April 2019	NA	−/−	Y
55 (1)	2400	FTF	Positive	1 day	15 January 2020	93	+/+	Y

^1^ Number of sows in the farm. ^2^ FTW: farrow-to-wean; FTF: farrow-to-feeder. ^3^ PRRSV history: negative (never infected with PRRSV) or positive (infected with PRRSV) farm. ^4^ Age of sampling. ^5^ Y = yes/N = no. NA = non-applicable because the farm is PRRSV-negative.

**Table 2:** Nursery farm and sample characteristics to study the agreement between paired serum and tongue samples (between brackets) from piglets belonging to the same production batch.

Farm (Pair)	Farm Size ^1^	Type of Farm ^2^	PRRSV History ^3^	Age ^4^	Sample Date	Timing from Diagnosis to Sampling	PRRSV Batch Results Serum/Tongues	Agreement (Y/N) ^5^
1 (1)	2500	WTF	Positive	8 weeks	4 April 2019	64	+/−	N
2 (1)	2520	WTFe	Naive	8 weeks	25 March 2019	NA	−/−	Y
4 (1)	2350	WTFe	Positive	8 weeks	5 February 2019	369	+/+	Y
4 (2)	2350	WTFe	Positive	8 weeks	12 March 2019	406	+/+	Y
4 (3)	2350	WTFe	Positive	8 weeks	9 May 2019	464	+/+	Y
5 (1)	3050	WTFe	Positive	8 weeks	4 February 2019	428	−/+	N
8 (1)	6000	WTFe	Positive	8 weeks	19 February 2019	120	+/+	Y

^1^ Number of nursery piglets in the farm^. 2^ WTFe: wean-to-feeder; WTF: wean-to-finish. ^3^ PRRSV history: negative (never infected with PRRSV) or positive (infected with PRRSV) farm. ^4^ Age of sampling. ^5^ Y = yes/N = no. NA = non-applicable because the farm is PRRSV negative.

## Data Availability

The study did not report any data.
